# Role of breast cancer inhibitors on diabetes mellitus– an *in silico* approach

**DOI:** 10.1186/s40200-015-0138-1

**Published:** 2015-03-17

**Authors:** Shailima DV Rampogu

**Affiliations:** Department of Biochemistry, Cachet Labs, Hyderabad, Telangana, India

**Keywords:** Cancer, Diabetes mellitus, 1ADS, Molegro, MVD, Breast cancer inhibitors

## Abstract

**Background:**

Breast cancer and diabetes mellitus type-2 are two of the major diseases, which cause death to the women worldwide. Around 16% of the breast cancer patients also suffer from diabetes mellitus. It has been noted that the risk of occurrence of the breast cancer is more in patients suffering from diabetes mellitus.

**Methods:**

Owing to this, the present article deals with an objective of using the breast cancer inhibitors for the diabetes drug target– 1ADS. Ten breast cancer inhibitors were drawn using Marvin Sketch. The docking was performed using Molegro software (Molegro Virtual Docker, MVD).

**Results:**

The docking wizard generated 50 poses with the highest Mol Dock score −131.649.

**Conclusion:**

This investigation successfully evaluated the effect of breast cancer inhibitors on diabetes mellitus, providing a new dimension in endocrine research.

## Introduction

Today, cancer and diabetes are two of the major global health risks [1,2]. There are several reports delineating how diabetes mellitus triggers a variety of cancers [3-8]. By the year 2007, in the United Sates alone, it was noted that about 8% of the total adult population were diabetic and 2.5 million people were reported having breast cancer [9,10]. Therefore, it is important to probe the relationship between diabetes mellitus and breast cancer The epidemiological studies have reported that the insulin-like growth factor 1 evokes the growth of colon tumour, suggesting the tumour growth is associated with the levels of insulin [11-15]. Knowing the fact that diabetes mellitus provokes the tumour cells, it would be ideal to design drugs that can act on both the diseases. One of such drugs is Metformin [2], which is a known diabetic agent acts by reducing the increased levels of insulin in blood [2]. Metformin decreases hyperglycaemia and hyperinsulinemia by increasing skeletal muscle glucose uptake [2]. Increased levels of insulin-like growth factor 1 (IGF-1) in serum/plasma are seen in prostate cancer and pre menopausal breast cancer [16-20].

**Figure 1:**
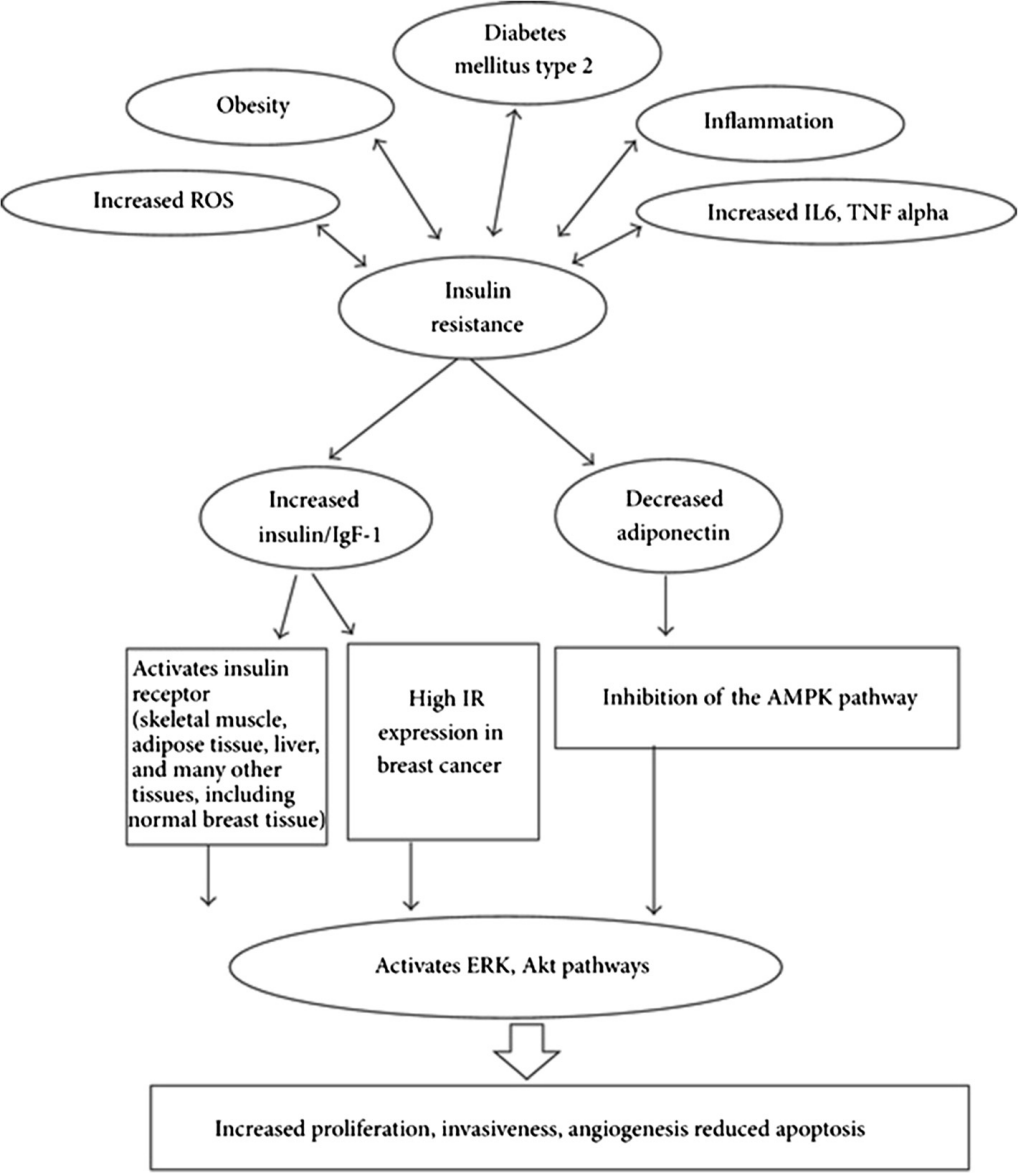
**Pathophysiological link between Diabetes and Breast Cancer** [22]**.**

The association of diabetes and breast cancer is a long debated topic. Among the cancers present today, breast cancer is considered to be one of the causes of deaths seen in women [21]. It was noted in 16% of the patients who suffer from breast cancer were found to be diagnosed with diabetes [22]. Diabetes brings about several modifications in hormonal system [22] which could trigger the enhancement of breast cancer like growth factors estrogen [22] and other growth factors. Diabetes inhibits the AMP kinase by decreasing the adiponectin plasma levels, which activates certain pathways like Akt and ERK aggravating the breast cancer risk Figure 1 [23,24].

**Figure 2:**
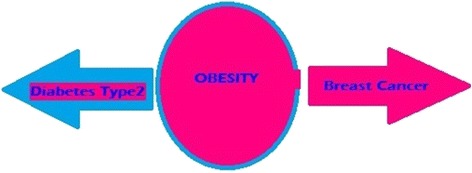
**Depiction between obesity and breast cancer.**

Breast cancer is seen in association with type 2 diabetes in women who have reached their post menopausal stage. In most of the cases it was observed that the overweight/obese women are more likely to develop both diseases as depicted in Figure 2.

1 ADS is the protein of interest in the present investigation. The elevated levels of the enzyme aldose reductase are seen associated with diabetes nephropathy [25]. There are reports which states the action of the aldose reductase inhibitors, which could control the diabetes prognosis and further its complications [26,27]. This leads to a very important notion of the use of cancer drugs on diabetes. P.Vigneri et al. reported the use of cancer drugs which favour the diabetes [28]. Hence, in the present article the breast cancer inhibitors are used to bind with the aldose reductase (1ADS).

Owing to this, the aim of the present article is to use the validated breast cancer inhibitors against the validated diabetic drug target protein via *in silico* analysis.

## Materials and methods

### Ligand preparation

**Figure 3:**
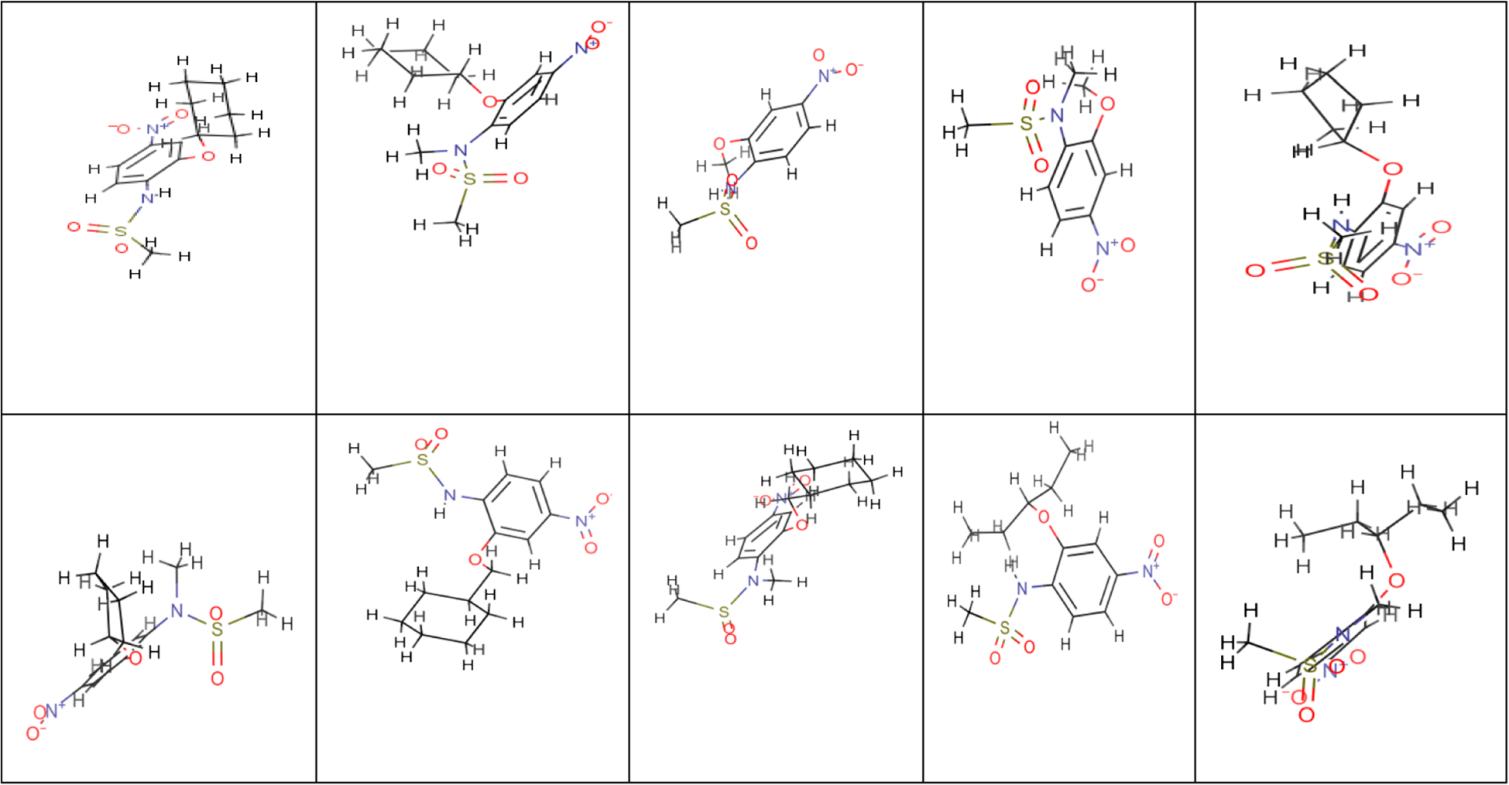
**Breast cancer inhibitors.**

The ligands used for the present study are validated breast cancer inhibitors [29]. Ten selected ligands were drawn using Marvin sketch, Figure 3 and subsequently imported into the Molegro.

### Protein selection and preparation

The protein selected for the present study was 1ADS at the resolution 2.40 Å. The protein from the Protein Data Bank (PDB) was downloaded and imported into Molegro. The protein and the ligands were prepared assigning “always” to the given parameters.

### Docking

**Figure 4:**
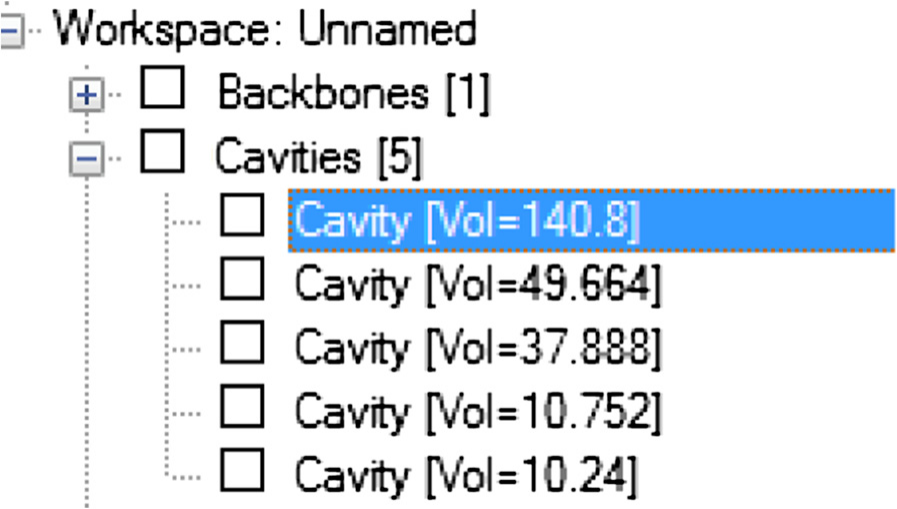
**Volume of the active sites.**

The docking was done between the diabetes drug target and the breast cancer inhibitors on the Molegro docking wizard. The active sites of the protein were identified and the site with highest area and volume was selected for the docking, Figure 4.

## Result

### The Molegro docking wizard revealed the following

**Figure 5:**
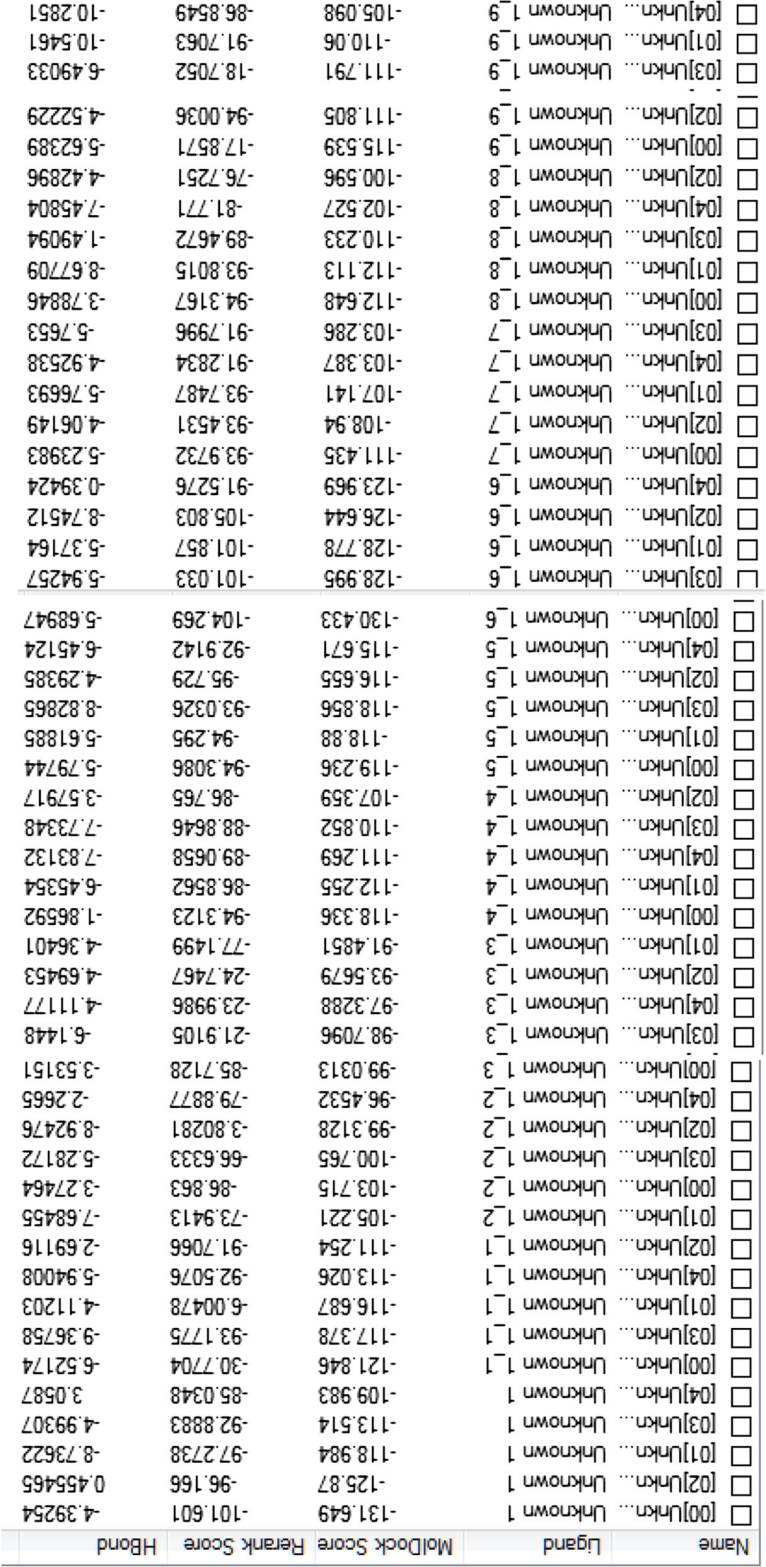
**Docking results.**

**Figure 6:**
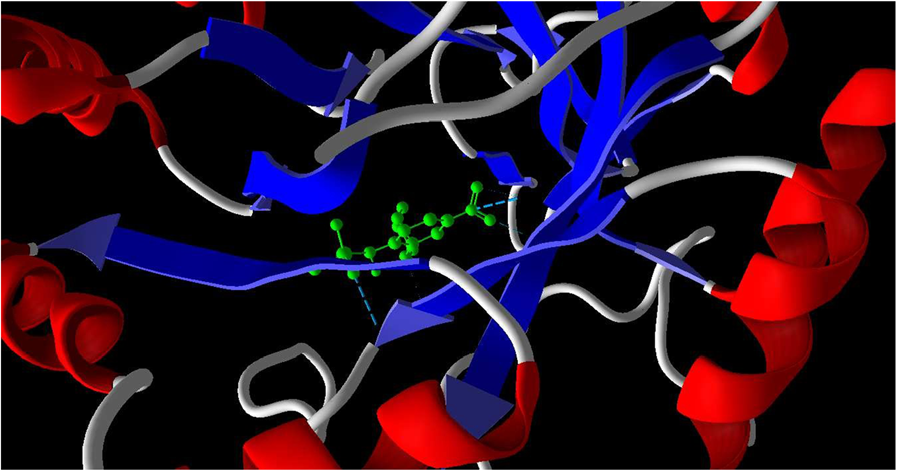
**Docking result showing hydrogen bonds.**

The docking wizard generated 50 poses Figure 5. The highest dock score, −131.649 was seen with the inhibitor 1. Hydrogen bond interactions were seen between the following amino acids Cys 298, Ser 210, Ser 159 and Asn 160 Figure 6.

## Discussion

Diabetes mellitus is one of the major metabolic disorders, which is associated with several other complications like nephropathy, vascular diseases and hypertension [30]. Besides this, diabetes mellitus initiates tumour formation at most of the sites [31] associated with some cancers such as breast cancer [32] and prostate cancer [31]. Reports also indicate the association of diabetes with pancreatic cancer [33] Hence it is important to address the problem and to evaluate the relationship between them. The present article has successfully evaluated the use of the breast cancer drugs on 1ADS, a diabetic drug target. This is a novel approach through which both the morbidities can be reduced.

The 10 breast cancer inhibitors considered for the present study were proven to be effective against aromatase, an enzyme seen in the breast cancer patients [21]. As breast cancer is seen commonly in the post menopausal diabetic women it is ideal to use a drug which could treat both the aliments. Encouraged by this fact, the breast cancer drugs are used against diabetes drug target, unlike vice-a verse as previously described in this article. The results show a new dimension in the research for controlling both the diseases, hence can reduce both the morbidities.

## Conclusion

Breast cancer and diabetes mellitus happen to be two of the major growing health concerns in the present day. Though not directly, diabetes mellitus is one of the causes of breast cancer incidences. It favours the growth of certain cancers like prostate cancer with a risk to other organs as well [28]. The present article successfully evaluates the role of breast cancer inhibitors on diabetes drug target. Given the best clinical attention, the associated relationship between both the diseases can be established, thus can cure both diseases.
